# An overview on the role of dietary phenolics for the treatment of cancers

**DOI:** 10.1186/s12937-016-0217-2

**Published:** 2016-12-01

**Authors:** Preethi G. Anantharaju, Prathima C. Gowda, Manjunatha G. Vimalambike, SubbaRao V. Madhunapantula

**Affiliations:** 1 Department of Biochemistry, Center of Excellence in Molecular Biology and Regenerative Medicine, JSS Medical College, JSS University, Mysore, 570 015, Karnataka India; 2 Department of Pharmacology, JSS Medical College, JSS University, Mysore, 570 015 Karnataka India; 3 Department of Pathology, JSS Medical College, JSS University, Mysore, 570 015 Karnataka India

**Keywords:** Tumor growth, Phenolic compounds, Anti-cancer activity, Cinnamic acid, Benzoic acid, Gallic acid, Caffeic acid

## Abstract

Plant derived phenolic compounds have been shown to inhibit the initiation and progression of cancers by modulating genes regulating key processes such as: (a) oncogenic transformation of normal cells; (b) growth and development of tumors; and (c) angiogenesis and metastasis. Recent studies focusing on identifying the molecular basis of plant phenolics-induced cancer cell death have demonstrated down-regulation of: (a) oncogenic survival kinases such as PI3K and Akt; (b) cell proliferation regulators that include Erk1/2, D-type Cyclins, and Cyclin Dependent Kinases (CDKs); (c) transcription factors such as NF-kβ, NRF2 and STATs; (d) histone deacetylases HDAC1 and HDAC2; and (e) angiogenic factors VEGF, FGFR1 and MIC-1. Furthermore, while inhibiting oncogenic proteins, the phenolic compounds elevate the expression of tumor suppressor proteins p53, PTEN, p21, and p27. In addition, plant phenolic compounds and the herbal extracts rich in phenolic compounds modulate the levels of reactive oxygen species (ROS) in cells thereby regulate cell proliferation, survival and apoptosis. Furthermore, recent studies have demonstrated that phenolic compounds undergo transformation in gut microbiota thereby acquire additional properties that promote their biological activities. In vitro observations, preclinical and epidemiological studies have shown the involvement of plant phenolic acids in retarding the cancer growth. However, to date, there is no clinical trial as such testing the role of plant phenolic compounds for inhibiting tumor growth in humans. More over, several variations in response to phenolic acid rich diets-mediated treatment among individuals have also been reported, raising concerns about whether phenolic acids could be used for treating cancers. Therefore, we have made an attempt to (a) address the key structural features of phenolic acids required for exhibiting potent anti-cancer activity; (b) review the reported findings about the mechanisms of action of phenolic compounds and their transformation by gut microbiota; and (c) update the toxicological aspects and anti-tumor properties of phenolic compounds and extracts containing phenolic compounds in animals.

## Introduction

Phenolic compounds are secondary metabolites in plants with a common aromatic ring bearing one or more hydroxyl groups [1]. More than 8000 natural phenolic compounds have been identified to date [2]. Phenolic compounds isolated from plant sources include simple phenols, flavonoids, lignins and lignans, tannins, xanthones, and coumarins [3]. These phenolic compounds are known to exhibit potent anti-cancer activities as well as combat various diseases associated with oxidative stress [4]. Prior studies have demonstrated that the health beneficial effects of dietary phenols are due to their ability to exhibit antioxidant, anti-inflammatory and anti-clastogenic activities [5]. Anti-carcinogenic effects of phenolic compounds is primarily due to the ability to: (a) induce cell cycle arrest; (b) inhibit oncogenic signaling cascades controlling cell proliferation, angiogenesis and apoptosis; (c) modulate ROS levels; (d) promote tumor suppressor proteins such as p53; and (e) enhance the ability to differentiate and transform in to normal cells etc. In this review article, we have discussed the anti-carcinogenic effects of simple phenolic compounds while addressing the mechanistic basis of tumor inhibition and various toxicological issues associated with the use of phenolic compounds.

### Classification of plant phenolics

**Fig. 1:**
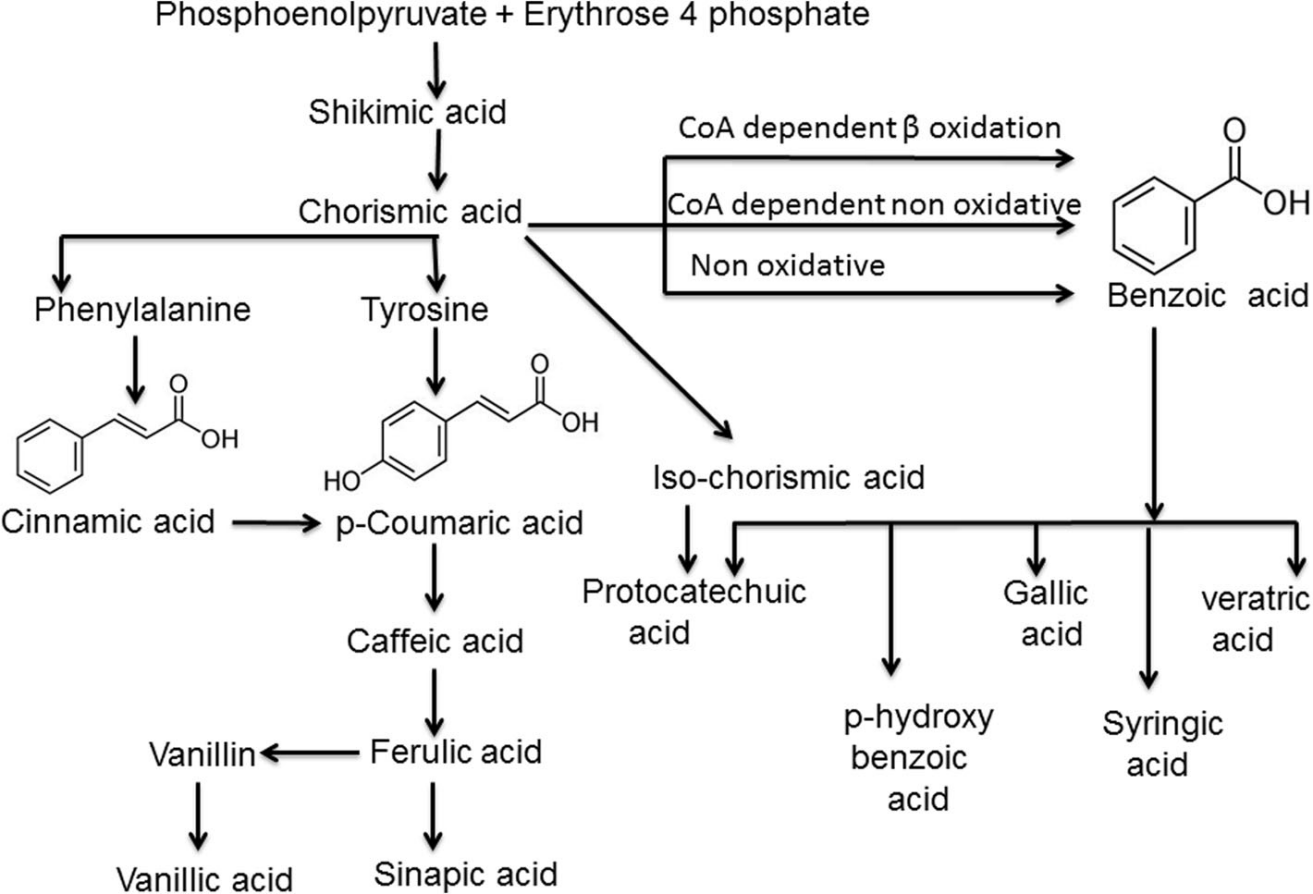
Synthesis of plant phenolic compounds by shikimate pathway, shows the biosynthetic pathway of plant phenolic acids [135]. The phosphoenolpyruvate react with erythrose-4-phosphate to give chorismic acid, which is a precursor for tyrosine and phenylalanine which later serves as precursors for cinnamic acid derivatives. Predominantly benzoic acids are synthesized from chorismic acid via oxidative and non-oxidative pathways while iso chorismic acid serve as precursor for protocatechuic acid [136]

**Fig. 2:**
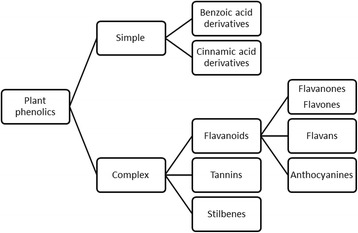
Classifications of the plant phenols, based on their structure. Broadly plant phenolic compounds are classified as simple and complex phenolic acids. Simple phenolic acids are further classified as benzoic and cinnamic acid derivatives while complex phenolic acids are classified as flavonoids, tannins and stilbenes

Phenylalanine and/or tyrosine are the precursors for the synthesis of phenolic acids through shikimate pathway [6] (Fig. 1). Addition of hydroxyl groups into the phenyl ring is the key step involved in the biosynthesis of phenolic acids [6]. Due to the heterogeneous structures of these phenolic acids, which range from low molecular weight single aromatic ring structure to high molecular weight polymeric compounds, they can be broadly classified into simple and complex phenolics (Fig. 2), which are discussed in detail in the following sections.

#### Simple Phenolics

Benzoic acids (BA) and cinnamic acids (CA) are the most simple phenolic acids found in nature with 6- and 9- carbon skeletons [7]. These compounds contain a carboxylic group attached to the benzene ring with one or more hydroxyl or methoxyl groups attached to it [8]. For example, gallic acid has three hydroxy (-OH) groups attached at 3rd (meta), 4th (para) and 5th (meta) carbon, whereas the syringic acid contain two methoxy (-OCH_3_) groups at 3rd and 5th (meta) and one -OH group at 4th carbon (para) [9]. The cinnamic acids in addition, have a unsaturated propionic acid side chain attached to the benzene ring [10]. Caffeic acid contains two -OH groups at 3rd and 4th carbon while Ferulic acid has one -OCH_3_ and one -OH group attached at 3rd and 4th carbon atoms respectively. Structures of benzoic and cinnamic acid derived phenolic compounds that are most commonly found in plants are shown in Table 1.

**Table 1:** Structures of benzoic and cinnamic acid derivatives commonly found in plants

Common name	Structure	IUPAC name	Molar Mass in g/mol	Primary source	Ref
Benzoic acid		Benzoic acid	122.12	Cranberries, Plums	https://caloriebee.com/nutrition/Effects-of-Benzoic-Acid-and-Benzoates-in-Food-and-Medicines
				Marachino cherries and Apples	
Cinnamylic acid		3-phenylprop-2-enoic acid	148.16	Cinnamon oil	[137]
*p*-Hydroxybenzoic acid		4- Hydroxy benzoic acid	138.12	Acia oil Green tea wine	[135]
p-Coumaric acid		3-(4-hydroxyphenyl)-2-propenoic acid	164.16	Barley grains Honey	[138, 139]
Protocatechuic acid		3,4-Dihydroxybenzoic acid	154.12	Plums Grapes	[75]
Caffeic acid		3-(3,4-Dihydroxyphenyl)-2-propenoic acid	180.16	Thyme, Oregano and sage	[21]
Gallic acid		3,4,5,Trihydroxy Benzoic acid	170.12	Chestnut Green chicory	[20]
Vanillic acid		4-Hydroxy-3-methoxybenzoic acid	168.14	Vinegar Wine	[140]
Isovanillic acid		3-hydroxy-4 methoxybenzoic acid	168.15	Saffron	[141]
Syringic acid		4-hydroxy-3,5-dimethoxybenzoic acid	198.17	Finger millet clove	[100]
Ferulic acid		3-(4-hydroxy-3-methoxy-phenyl)prop-2-enoic acid	194.18	Barley	[142]
Veratric acid		3,4 Dimethoxy Benzoic acid	182.17	Not available	
Chlorogenic acid (3-Caffeoylquinic acid)		3-{[(2E)-3-(3,4-dihydroxyphenyl)prop-2-enoyl]oxy}-1,4,5-trihydroxycyclohexanecarboxylic acid	354.31	Green coffee seeds	[143]
Di-caffeoylquinic acid (Cynarine)		(1*R*,3*R*,4*S*,5*R*)-1,3-bis[[(*E*)-3-(3,4-dihydroxyphenyl)prop-2-enoyl]oxy]-4,5-dihydroxycyclohexane-1-carboxylic acid	516.46	Artichoke	[144]

Table 1 shows the structures, common and IUPAC names along with the molecular mass of commonly found BA and CA derivatives. References that have discussed about these phenolic compounds are also listed

#### Complex Phenolics

Complex phenolics are compounds with higher molecular weight. These phenolic acids are most commonly found in cell vacuoles [11]. Tannins and flavonoids are the best examples for complex phenolics found among fruits and vegetables [12]. Flavonoids are made up of 2 phenolic rings to which an oxygenated heterocyclic pyran ring is attached [13]. The oxygenated status of the pyran ring further classifies flavonoids into: anthocyanins, flavones, flavanols etc. Higher complexity is achieved due to the acetylation or glycosylation of these molecules beyond their primary substitutions with hydroxyl or methoxyl groups [14]. Since the focus of this review article is on simple phenolic compounds the details pertaining to complex phenolic compounds is not much discussed. For detailed structural and functional aspects of flavonoids and their derivatives the reader can refer recent review articles as shown in references [2, 13, 15].

### Distribution of phenolics in various parts of the plants

Phenolic acids are widely distributed among various parts of the plants including roots, leaves, fruits and vegetables [16]. The caffeic acid is the most common type of phenolic acid found widely in the fruits, while the ferulic acid is found in the cell walls of seed coat, bran and fruits in esterified form [17]. The leaves and stems of plants contain higher levels of phenolic acids with significant variations found among different species [16]. For instance, complex polyphenols are found in the cell vacuoles, tissues present in the leaf, epidermis, flowers and fruits [12]. Likewise, barks, wood and fruit pods are rich in tannins, while flowers contain more flavonoids [18].

### Dietary sources

Plant phenolic acids form an integral part of our diet and thus making them a prime focus of interest. For example, cereals, legumes, soybeans, coffee, tea, rosemary and thyme, which are widely utilized in diet, are the rich sources of phenolic acids [19]. A study has shown that gallic acid is the most common phenolic acid found among fruits, vegetables, chestnuts and green chicory [20]. While a separate study by Kivilompolo M 2007 et al., reported that herbs like thyme, oregano and sage are rich in caffeic acids [21], another study by J Perez-Jimenez 2010 et al., identified polyphenolic compounds in the seasonings such as star anise, cloves, peppermint and celery seeds [22]. Fruits from berry and citrus family members, apples and pears have also known to contain considerable amounts of phenolic acids [12]. Among vegetables the yellow onions followed by artichokes, potatoes, rhubarb, red cabbage and cherry tomatoes harbor high phenolic content [23, 24]. Among the different vegetables consumed, the potatoes account for up to 25% of total phenolics such as chlorogenic acid and caffeic acid [25].

### Nutritive value

Phenolic acids are the major essential non-nutrients present in the diet. However, most often, they are considered as antinutrients due to the adverse effects of tannins on protein digestibility [26]. But, in recent years, an increased interest in developing diets rich in plant derived polyphenols is observed due to the realization that the phenolic compounds exhibit anti-oxidant, anti-inflammatory and anti-proliferative effects [4]. Since these activities are essential for preventing or treating cancers, cardiovascular diseases, atherosclerosis and neurodegenerative disorders, supplementing diets with extracts rich phenolic compounds help to effectively manage these disorders [27]. For instance, polyphenols from green tea were shown to inhibit the progression of tumors in different animal models [28]. Similarly, a much lowered risk of colon, prostate and breast cancers observed among the Asian population was attributed to the intake of polyphenol and flavonoid rich diets [29]. In addition, a study carried out in Finland reported healthier carotid arteries with less obstruction from atherosclerosis when individuals consumed higher amounts of flavonoids and polyphenols [30]. Likewise, a separate study in France revealed that individuals consuming diets supplemented with flavonoid-based plant products had lesser incidence of cognitive disorders over the age of 65 years [31]. In summary, these studies highlight the importance of consuming diets with phenolic compounds in mitigating the disease severity.

### Metabolism of phenolic compounds by gut microbiota

**Fig. 3:**
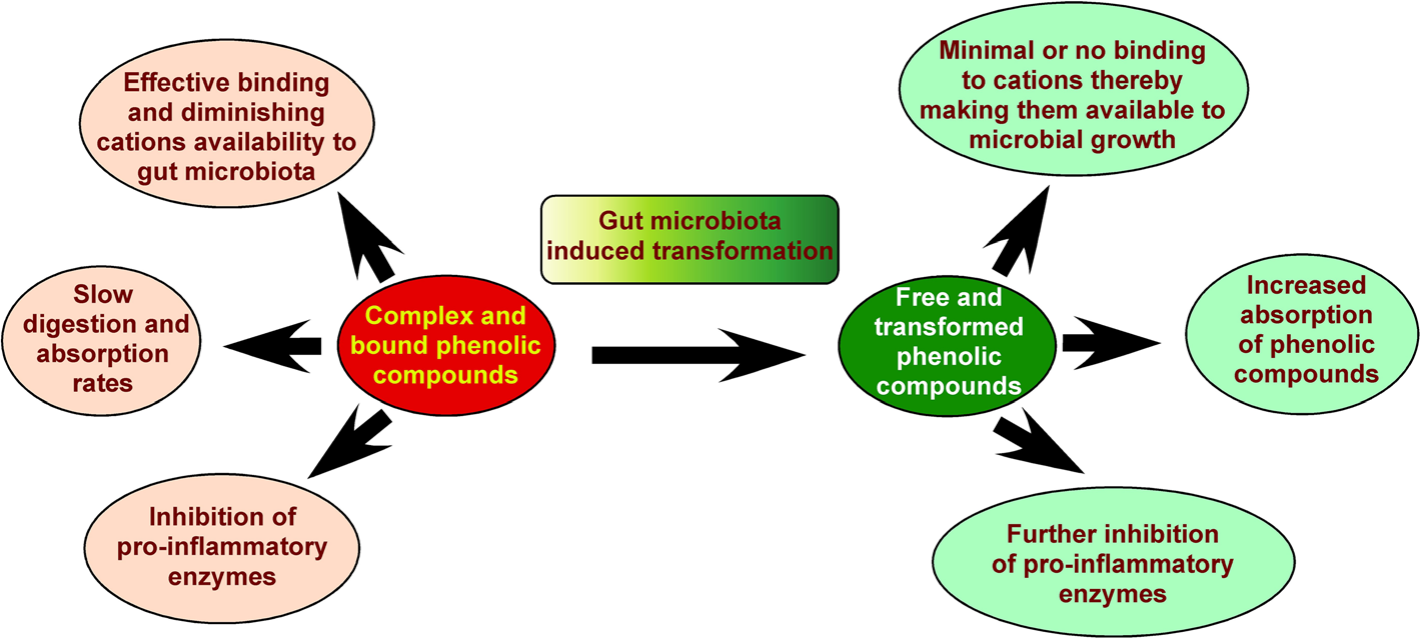
Gut microbiota mediates the transformation of phenolic compounds and enhance the health beneficial effects. Bound and complex phenolic compounds undergo bio-transformation in the human gastro intestinal tract by the gut microbiota into simple and easily bioavailable phenolic compounds. The active metabolites produced by the biotransformation have proven to exhibit better pharmacological actions and possess better health beneficial properties compared to corresponding untransformed compounds

The polyphenolic compounds present in nature are usually glycosylated and exist as complex molecules with poor solubility and less bioavailability [32]. Once ingested in diet, these complex insoluble phenolics undergo transformation in the human gastro-intestinal tract by the enzymes and microbiota to produce phenolic compounds with more bioavailability and better pharmacological properties [32]. For example, a recent study has demonstrated that ellagic acid, a phenolic acid derived from berries, is transformed by the gut microbiota in to urolithin. The bio-transformed urolithin effectively inhibits heme peroxidases such as myeloperoxidase and lactoperoxidase thereby reduce the inflammation mediated cellular damage [33]. In addition, urolithin reduces superoxide radicals’ generation while ellagic acid failed to do so [33]. In animal models oral administration of urolithin, at 40 mg/kg body weight, inhibited PMA-induced edema and MPO activity compared to ellagic acid [33]. A separate study by Marin et al., demonstrated the production of soluble phenolic compounds such as ferulic acid and coumaric acid by the hydrolytic activity of gut microbiota on ester linked arabinoxylans [32]. Additional studies have further shown that the dimers of ferulic acid are degraded by micro-organisms present in the gut in to vanillin and 3-(4-hydroxyphenyl)-propionic acid with enhanced anti-cancer activity [32]. Therefore, transformation of phenolic compounds by gut microbiota has several important roles and influences their biochemical properties as well as contribute for the variations in the response to treatment with phenolic compounds among individual [34] (Fig. 3).

### Anticancer activities of crude extracts/mixture of phenolic compounds

Purification of phenolic compounds from natural sources to homogeneity is a challenging task. Therefore, several studies have tested the ability of either crude extracts rich in phenolic compounds or the fractions containing a mixture of phenolic compounds for inhibiting cancers in vitro and in vivo. For example the extracts of Pandanus amaryllifolius (herbal plant from Malaysian region) containing gallic acid, cinnamic acid and ferulic acid are reported to inhibit breast cancer cell lines in vitro [35]. Similarly, the extracts of Baccharis trimera, containing gallic acid, pyrogallol, syringic acid and caffeic acid were shown to suppress the formation of tumor cell colonies and proliferation of SiHa cells in a dose dependent manner [36]. Likewise, Thanaset et al., 2014 have demonstrated the time- and dose dependent growth inhibitory effect of water soluble constituents of commercially available Houttuynia cordata fermentation products on HeLa, HCT116, and HT29 cells. Seven phenolic acids including protocatechuic, p-hydroxybenzoic, vanillic, syringic, p-coumaric, ferulic, and sinapic acids were identified in the water soluble fraction of H.cordata [37]. Another study isolated and characterized gallic acid from Toona sinensis leaf extracts which inhibited the growth of prostate cancer cell lines [38]. In a separate study Kurata et al 2007, could identify caffeic acid, chlorogenic acid, 3,4-di-O-caffeoylquinic acid, 3,5-di-O-caffeoylquinic acid, 4,5-di-O-caffeoylquinic acid, and 3,4,5-tri-O-caffeoylquinic acid in the leaves of the sweet potatoes inhibited the growth of stomach cancer (Kato III), a colon cancer (DLD-1), and a promyelocytic leukemia cell (HL-60) [39]. Thus it can be concluded that the antitumor activity exerted by these plant extracts could be due to the presence of phenolic acids. However, utility of crude extracts containing a mixture of phenolic compounds as drugs require more careful investigations as the proportion of each phenolic compound in the extract might vary from source to source as well as from the method of isolation and fractionation.

## Key structural features and mechanism of action of plant phenolics

**Fig. 4:**
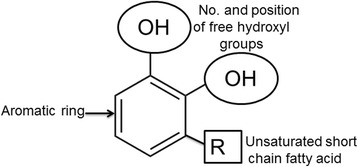
Key structural motifs responsible for anticancer activity Studies on the structure-activity-relationship (SAR) of phenolic compounds have identified the aromatic ring (represented with an arrow), number and position of free hydroxyl groups (represented with a circle) and unsaturated fatty acid chain as key structural motifs required for exhibiting anticancer activity (represented with square)

Even though phenolic compounds have been shown to exhibit anticancer activity, the efficacy varies from one compound to other, which is due to the variations in their structures as well as their molecular targets. For example, structure activity relationship studies delineating the key functional groups required for exhibiting potent anti-cancer effects of phenolic compounds have identified the involvement of aromatic ring and hydroxylic groups [40, 41], (Fig. 4). Compounds with more number of hydroxylic groups exhibited better anticancer activity compared to the ones with no hydroxylic groups or compounds with –OCH3 moieties. For instance, gallic acid that contains three hydroxylic groups is reported to be more effective compared to dihydroxy benzoic acid or monohydroxy benzoic acid. In addition to hydroxylic groups and catechol ring structure, the presence of an unsaturated short fatty acid side chain also makes the phenolic compounds more potent. As an example, studies comparing the efficacy of cinnamic acid and benzoic acid for inhibiting cancer cell growth revealed that cinnamic acids that contain a unsaturated propionic acid side chain are more better anti-cancer agents [40, 41]. Thus phenolic acids i.e benzoic and cinnamic acid derivatives with higher hydroxyl substitutions could be considered as potential candidates for preventing the cancer cell proliferation.

Benzoic acid derivatives such as gallic acid, protocatechuic acids that are commonly found in nature have attained much attention due to their better pharmacological properties [42–44]. For instance, hydroxybenzoic acids and protocatechuic acid have been reported to exhibit dose dependent cytotoxicity on prostate (PC-3) and breast (MCF-7) cancer cells [45]. Mechanistically, 4-hydroxy benzoic acid inhibited the histone deacetylase enzyme thus modifying the acetylation process thereby inducing the cancer cell death [46]. Protocatechuic acid targets the RhoB activation leading to decreased MMP thus inhibiting the cancer cell migration [44]. These compounds induce apoptosis, and enhance lactate dehydrogenase levels by reducing the mitochondrial membrane potential. In addition, these compounds trigger the fragmentation of DNA in breast, lung, liver and prostate cancer cell lines [47]. Similarly, gallic acid inhibits the cancer cell proliferation by promoting the generation of reactive oxygen species and arresting cells in G2/M phase [38]. Cinnamic acid derivatives CAPE- caffeic acid phenyl ester and Caffeic acid are also well known antioxidants, reported to modulate key signaling pathways such as NF- kβ, MAPK and AKT [48]. Furthermore, CAPE and CAA induced cell death via apoptosis and cell cycle arrest in cell lines representing carcinomas of oral cavity, neck and tongue [40, 49, 50]. In addition, CAPE is known to inhibit the nuclear factor kappa-light chain enhancer of activated B cells i.e NF-kβ, which further enhanced the expression levels of p21, p53 [51]. P-Coumaric acid, an abundant isomer of cinnamic acid, inhibits the colon cancer cell lines by increasing the ROS levels, decreasing the mitochondrial membrane potential and inhibiting the cell cycle at sub G1 phase [52]. Esters of cinnamic acids are more potent when compared to the hydroxylated or the methoxylated versions [40]. Among the 20 different caffeic acid analogues, 4-phenylbutyl caffeate, (*Z*)-8-phenyl-7-octenyl and (*E*)-8-phenyl-7-octenyl caffeate exhibited potent anti-tumor activity with EC_50_value of 0.02 μM [53].

## In vitro studies

### Effect on cell viability and proliferation

Uncontrolled cellular proliferation is a hall mark feature of cancer cells [54]. Therefore, the efficiency of anticancer drugs can be measured by their ability to: (a) decrease cell proliferation measured using the incorporation of tritiated Thymidine or Bromodeoxy Uridine; (b) attenuate the viability of cells as measured using MTT or SRB; (c) arrest the cells in interphase (G0/G1, S or G2/M) [55]. Phenolic compounds isolated from plant sources have been demonstrated to inhibit the proliferation of cancer cells [12]. For example, cinnamic acid reduced the viability of HT-44 melanoma cell line in a dose dependent manner with the IC_50_ of 2.4 mM [56]. Mechanistically, cinnamic acid decreased the number of S phase cells thereby inhibited the proliferation of HT-44 cells [56]. Likewise, a separate study showed the induction of cell cycle arrest at G2/M phase when breast cancer cells MDA-MB-231 and MCF-7 were exposed to 4-(3,4,5-Trimethoxyphenoxy) benzoic acid [57]. Similarly, many other studies have also demonstrated the efficacy of phenolic compounds to retard cancer cell proliferation [52]. For instance, p-coumaric acid decreased the viability of HCT-15 and HT-29 cells at a concentration of 1400 and 1600 μmol/L, respectively [52]. Reduction in the number of colonies formed in the coumaric acid treated HCT-15 (32) and HT-29 (51) against the untreated (105 and 154) at 72 h confirmed the anti-proliferative effects of these compounds [52]. Other phenolic acids that have shown to exhibit antiproliferative activity include: (a) Caffeic- and 5-caffeoylquinic acid that reduced the cell viability of HT-29 colorectal carcinoma cell line and HT-1080 fibrosarcoma cells at 96 h by modulating cell cycle stages [58, 59]; (b) Di-caffeoylquinic acid, isolated from sweet potato, which inhibited the proliferation of DLD-1 human colon cancer cells [39]; (c) Ferulic acid and p-Coumaric acid that were shown to inhibit the pancreatic cancer cell line MIA-Pa-Ca-2 [60]; (d) Gallic acid, which inhibited cervical carcinomas cells HeLa and HTB-35 and Jukart [61, 62]. Similarly, even the phenyl substituted cinnamic acids also exhibited significant cytotoxicity in HT-29, A-549, OAW-42, MDA-MB-231 and HeLa cell lines with IC_50_ values of <60 μM [63]. Efficacy evaluation studies comparing the potency of phenyl substituted acids reported that at 0.1 mM concentration ~84–92% cells were killed compared to the non-substituted derivatives, which had an effect of ~20–63% [63]. The IC_50_ values of phenolic compounds are tabulated in Table 2. In conclusion both cinnamic and benzoic acid derivatives possess potent anti-proliferative activity as evident by the low IC_50_ values exhibited by them on several cancer cell lines.

**Table 2:** Reported anti-cancer activities of plant phenolic acids

Phenolic Acid/Phenolic Acid Derivative	Cancers Tested	Cell Lines Used	Reported IC_50_ (μM)	Reference
Cinnamic acid	Melanoma	HT-144	2400.0	[56]
	Colon	HT-29	1000.0	[91]
Caffeic acid phenyl ester	Prostate	LNCaP	33.0	[145]
	Breast	MDA-MB-231	14.0	[146]
		Hs578T	8.0	
p-Coumaric acid	Neuroblastoma	N2a	104.0	[147]
	Breast	T47D	474.0	[148]
	Colon	SW-620	87.0	[148]
	Liver	HepG2	215.0	[148]
	Lung	A549	412.0	[148]
Ferulic acid	Prostate	PC-3	300.0	[149]
		LNCaP	500.0	
	Pancreatic	MIA PaCa-2	500.0	[60]
Gallic acid	Colon	HCT-15	0.5	[150]
	Breast	MDA-MB-231	0.4	[150]

Table 2 summarizes the anticancer activity of the plant phenolic acids on various cell lines with their IC_50_ values. The IC_50_ values varied from nM to mM concentrations

### Effect on cellular apoptosis

Apoptosis, a suicidal cell death mechanism, is a well-controlled process that gets activated either by the intrinsic or the extrinsic pathways [64]. The cells undergoing apoptosis exhibit caspase mediated cell death through transforming pro-caspases into active caspases [64]. Whereas the extrinsic pathway gets activated when the binding of Fas ligands to Fas proteins, the intrinsic pathway is triggered by oxidative stress inside the cells releasing the cytochrome-C from the disrupted mitochondria [65]. A prior study reported the induction of caspase-9 when HT-44 cells were treated with cinnamic acid [56]. Activated caspase-9 in turn activated other caspases such as Caspase-3 and -7 [56]. A separate study demonstrated that Ferulic acid could upregulate Bax and downregulate Bcl-2 to induce apoptosis in osteosarcoma cells [64]. Similarly, FA has induced the expression of Bax, caspase-3 and -9 in fibrosarcoma cells [60]. Elevated caspase-3 was also observed when breast cancer cells were treated with 4-(3,4,5-Trimethoxyphenoxy) benzoic acid [57]. Propidium iodide staining of cells followed by analyzing the stained cells with flow cytometer is another way of measuring the apoptotic cell death [66]. Elevated subG0/G1 population is a clear indicator of apoptosis induced by pharmacological agents promoting DNA damage [67]. A recent study showed that colon cancer cells treated with p-coumaric acid have undergone apoptosis as evidenced by a significant increase in the subG1 phase cell population [52]. Further confirming this data, analysis of cellular images captured by scanning electron microscope (SEM) exhibited typical signs of apoptosis such as membrane blebbing and shrinkage, which were absent among the normal cells [52]. Likewise, Protocatechuic acid also elevated the caspase-3 levels and reduced the membrane potential in breast, prostate, lung, cervical and hepatic cell lines in a dose dependent manner [47]. Similarly, a dose dependent increase in the caffeic acid concentration resulted in an increase in the apoptosis in human HT-1080 fibrosarcoma cell line [59]. Induction of apoptosis by caffeic acid was confirmed by the morphological changes and the acridine orange and ethidium bromide staining methods [59]. In summary, the induction of apoptosis is a major phenomenon exhibited by phenolic compounds.

### Effect on cellular migration

Tumor cell migration and invasions are the strategies developed and adopted by cancer cells to localize into distant organs and to escape drug treatment [68]. Since metastatic tumor cells are hard to treat due to their ability to circulate in the blood as well as unusual expression of oncogenic proteins compared to primary tumors, better strategies are required to prevent the metastatic spread [54]. One such strategy is to use naturally occurring plant derived phenolic compounds, which are known to halt the migration of cells by interfering with epithelial-to-mesenchymal transition (EMT), cell invasion and extravasation [69]. For instance, ferulic acid suppressed metastasis in breast cancer cell lines by reversing the epithelial-mesenchymal transition (EMT) [69]. Similarly, 4-methyl-3-nitrobenzoic acid derivative inhibited the epidermal growth factor (EGF)-induced migration and chemotaxis of breast cancer cell lines [57]. Caffeic acid phenyl esters inhibited the metastasis of breast (MDA-MB-231 and MDA-MB-468), colon (SW620) and non-small cell lung cancer (H460) cell lines by blocking the voltage-gated sodium channels [70]. Recent studies have shown that voltage-gated sodium channels could modulate vascular endothelial growth factor (VEGF)-induced proliferation, tubular differentiation and adhesion by elevating the intracellular Ca^2+^, which activates PKC and ERK-1/2 [71]. However, in a separate study the caffeic acid phenyl esters was shown to retard migration of cells by 38, 56, and 82% at 24, 48, and 72 h, respectively [72]. Gallic acid, another simple phenolic compound, also increased the expression of RhoB thereby inhibiting the metastasis of gastric cancer cells [43]. In summary these phenolic acids inhibit tumor cell migration and invasions and thus localize the cancer cells thereby sensitizing them to drugs.

## In vivo studies

### Toxicity studies

Compared to many synthetic and semi-synthetic compounds, naturally occurring phenolic compounds are less toxic and safe even at higher doses [73]. However, before evaluating in the clinical trials, one has to determine the safety and toxicity profiles of phenolic compounds using animal models. Since some of the phenolic compounds are also known to induce the formation of tumors by transforming the normal cells, it is mandatory to study the safety and toxicity of these plant derived products [74]. Protocatechuic acid is another phenolic compound tested in mice for safety and toxicity. The LD_50_ of protocatechuic acid in mice was reported to be 800 mg/kg by i.p., and 3.5 g/kg by i.v. route [75]. However, when administered orally, the LD_50_ of protocatechuic aldehyde was about 1.7 g/kg [75]. Sub-chronic toxicity assessment of gallic acid, a well-known antioxidant benzoic acid derivative, in rats showed a relatively safe profile [76]. Experimentally, rats fed with gallic acid (0, 0.2, 0.6, 1.7 and 5%) containing diet for 13 weeks exhibited no toxic symptoms even at 119 and 128 mg/kg/day for male and female rats, respectively [76]. In mice, oral administration of a dose up to 5000 mg/kg was also found to be safe [77]. Compared to gallic acid, p-coumaric acid exhibited low toxicity with LD_50_ ~ 2850 mg/kg body weight [78]. In conclusion, the safety and toxicity profiles of phenolic compounds vary depending upon their structure, route of administration and the animals that are being studied. Therefore, it is recommended to determine the safety and toxicity profiles of phenolic compounds before moving them in to preclinical and clinical studies.

### Efficacy studies

Efficacy of a compound depends on its absorption, distribution, metabolism, excretion, availability, stability, route of administration, target specificity and ability to control key pathways regulating cancer cell proliferation [79]. A number of studies have reported variability in these properties for various plant phenolic compounds. Most of the soluble phenolic acids are rapidly absorbed in the small intestine while the insoluble esterified forms are absorbed in the colon [80, 81]. The phenolic acids are rapidly absorbed by the Monocarboxylic acid transporters (MCT) or through para-cellular diffusion [82], while, excretion is majorly through urine with only a low percent of it is through bile [83].

Phenolic compounds with good ADME properties are likely to exhibit potent anti-tumor activities [84]. For example, caffeic acid phenyl propyl ester (CAPPE) and caffeic acid phenyl ethyl ester (CAPE), which are the better bioavailable versions of caffeic acid, significantly inhibited the growth of colorectal tumors in xenografted mouse models by decreasing the number of proliferating cells as evident by reduced expression of proliferating cell nuclear antigen (PCNA), and by inhibiting fatty acid synthetase (FASN) as well as matrix metalloproteinase 9 (MMP-9) [85]. Likewise, a separate study showed that athymic mice harboring HepG2 xenografted tumors were susceptible to growth inhibition when treated subcutaneously (5 mg/kg, thrice a week up-to 10 days) or orally (20 mg/kg, daily up-to 6 weeks) with CAA and CAPE [86]. The data revealed that tumor size decreased significantly as evident by 61 and 56.7% inhibition among the CAA and CAPE subcutaneously treated groups respectively [86]. However, when these compounds were administered orally, the reduction was around 53.6 and 47.1%, respectively with caffeic acid and caffeic acid phenyl ester groups [86]. Similarly, 10 mg/kg of CAPE administered intraperitoneally inhibited the development of cholangiocarcinoma [87]. Anti-cancer activity of other phenolic compounds is also well explored [33, 80, 81]. For instance, a recent study has shown that oral administration of protocatechuic acid at a dose of 20 and 40 mg/100 g reduced the metastatic nodule formation in C57/BL6 mice [44]. In a similar fashion, ferulic acid at a dose of 10, 30 and 50 mg/kg body weight inhibited the melanoma tumor development in nude mice [88]. The trihydroxy benzoic acid (gallic acid at 0.3 and 1.0%) also inhibited the growth of osteocarcinomas in xenograft mice models [89]. The data showed a significant reduction in the tumor volume from 2392.99 mm^3^ in control group to 1896.34 mm^3^ and 1476.55 mm^3^ in the treated groups [89]. Another study also demonstrated the anti-tumor potential of gallic acid. Mice fed with 0.3 and 1% gallic acid for 20 weeks inhibited the prostate cancer growth and progression to advanced stages [90]. Even the derivatives of phenolic compounds also exhibited anti-cancer activity, which is either similar to- or much better than parent underivatized compound. A study reported that 4-methyl-3 nitro benzoic acid derivative along with paclitaxel synergistically inhibited the metastasis of breast cancer in SCID xenograft mice models [57]. Zhu, B. et al showed that intragastric administration of cinnamic acid suppressed the colon cancer in athymic mouse at a well-tolerated dose 1.0 and 1.5 mmol/kg [91]. Irrespective of the mode of administration, these phenolic acids are capable of inhibiting the tumor growth in-vivo.

## Mechanism of action

### Effect on ROS generation

Reactive oxygen species such as hydroxyl radicals, hydrogen peroxide and superoxide radicals are generated on a regular basis during the mitochondrial respiration [92]. Lower levels of ROS is involved in regulating several signaling pathways that include cell survival, cell proliferation, metabolism, anti-oxidant and anti-inflammatory responses, iron homeostasis etc. [93]. Moderate increase in the ROS levels is suppressed by the anti-oxidant chemicals or proteins present in the cells [94]. Therefore, in general, normal cells maintain a fine balance in terms of ROS level. However, under certain conditions the levels of ROS exceeds the anti-oxidant defense capacity leading to oxidative stress [95]. The free radicals produced in cells during oxidative stress are highly reactive and capable of inducing tissue damage by reacting with membrane lipids, nucleotides, sulphydryl groups of proteins and by cross-linking/fragmentation of ribonucleoproteins [96]. As a result of these ROS induced insults, the cells undergo transformation and form a variety of tumors [97]. ROS generated in the cancer cells are involved in activation of several transcription factors including the NF-kβ, STAT3, activator protein-1 etc. which is essential in controlling cellular proliferation, tumor survival, angiogenesis etc. [98]. In addition to DNA damage, ROS can induce alkali labile sites, single strand breaks and damages to purines and pyrimidines [98]. Although, tumor cells have higher levels of ROS than the normal cells; elevated levels beyond the threshold for prolonged duration can induce damage in cancer cells [52]. Therefore, promoting ROS generation in cancer cells is a viable strategy to inhibit tumor growth. For example, cinnamic acid derivative p-coumaric acid triggered cancer cell death via increasing the levels of ROS [52]. Likewise, a reduction in the mitochondrial membrane potential in the colon cancer cell HCT-15 was reported when cells were exposed to caffeic acid [99]. Thus elevating the ROS levels by treating with phenolic compounds is a viable strategy to control cancer cells proliferation.

### Effect on Oncogenic pathways

Mutations in the proto-oncogenes leads to formation of oncogenes, which code for proteins that help cancer cells proliferate and survive even in the conditions that are not hostile [100]. For instance, oncogenic mutations in B-Raf kinase have been reported in melanomas, colorectal, and ovarian cancers [101]. Mutant ^V600E^B-Raf is constitutively active and doesn’t require the translocation and association with Ras proteins for enzyme activity [102]. As a result, cells harboring this protein proliferate in an uncontrolled fashion, which ultimately leads to the formation of malignant metastatic tumors [103]. Prior reports evaluating the efficacy of targeting mutant oncogenic proteins such as ^V600E^B-Raf have shown decreased tumor growth in preclinical animal models as well as in Phase-III clinical trials [104]. Therefore identifying natural products such as phenolic compounds that could effectively inhibit B-Raf kinase signaling are the potential candidates for developing clinically viable drugs [105, 106].

Recently, caffeic acid has shown to inhibit the metastasis of colon cancer by inhibiting the phosphorylation of Extracellular signal Regulated Kinases (ERK) which is a downstream target of Raf [107].

**Fig. 5:**
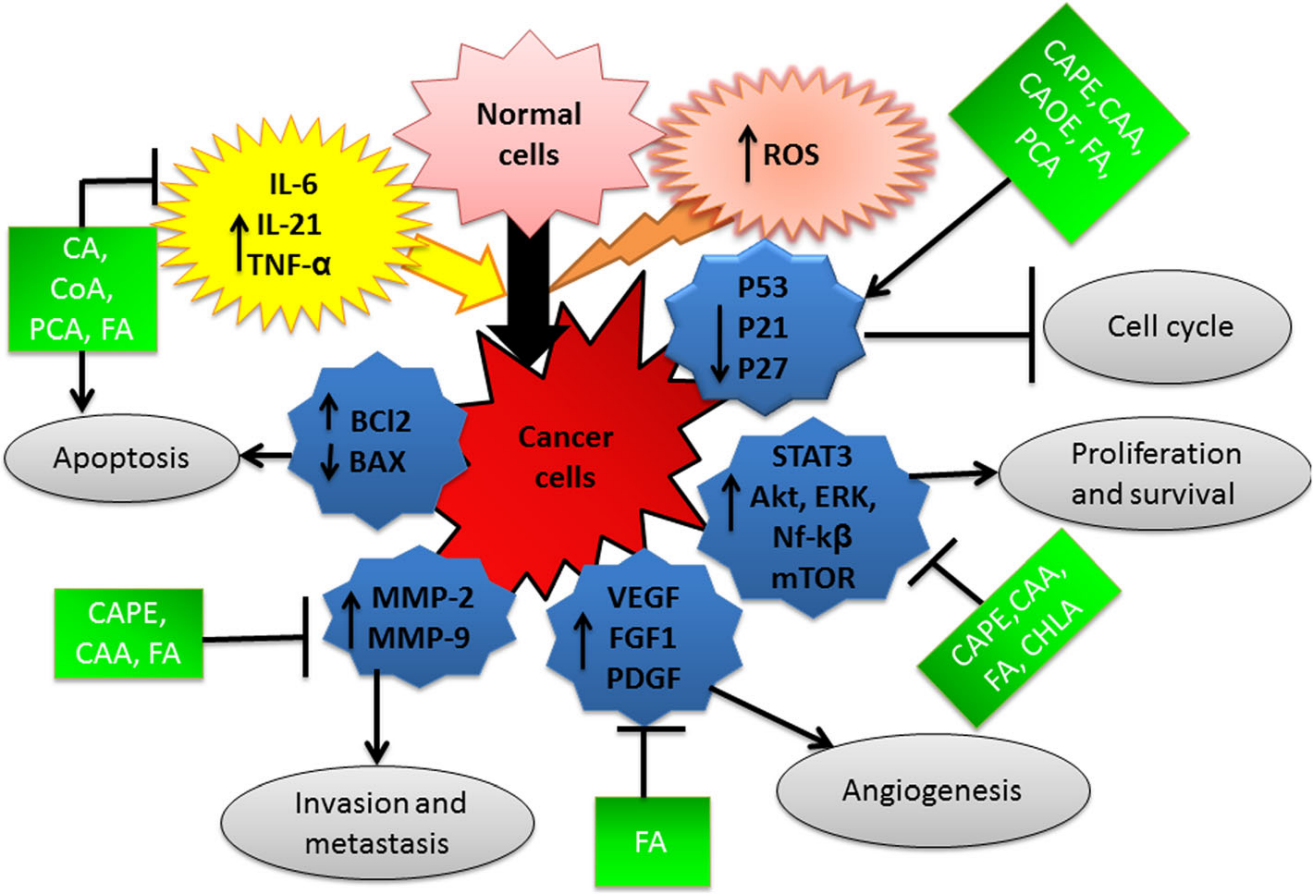
Key processes effected by plant phenolic acids Phenolic compounds are known to modulate the expression of key proteins such as BCL_2_, BAX, AKT, STAT. While suppressing the oncogenic proteins, phenolic compounds induce the expression of tumor suppressors P53, P21 and P27. As a result, phenolic compounds trigger apoptotic cell death while inhibiting the proliferation and inducing the cell cycle arrest [47]

In addition, caffeic acid has been demonstrated to inhibit LPS-induced oxidative stress by retarding the ERK signaling in endothelial cells [108]. Likewise, protocatechuic acid inhibited NF-kβ and MAPK signaling cascades to down-regulate the proliferation of lung and gastric carcinoma cells [44, 109]. Similarly, even the ferulic acid and caffeic acid phenyl ester also down regulated phosphorylated PI3K and AKT signaling cascades to inhibit melanoma cells proliferation as well as to induce apoptosis [88, 110]. Furthermore, in addition to downregulating the phosphorylation of Akt kinase, Caffeic acid phenyl ester has suppressed the expression of AKT isoforms in prostate cancer cells [111]. Few other studies have also reported the therapeutic efficacy of cinnamic acid derivatives such as ferulic acid, caffeic acid and chlorogenic acids for treating cancers [112]. The data revealed that these cinnamic acid derivatives down modulate MAPK and AKT signaling pathways to inactivate the NF-kβ, AP-1 and STAT3 in lung adenocarcinomas [112]. Thus phenolic acids are known to play a crucial role in blocking the oncogenic pathways to inhibit cancer progression as shown in Fig. 5.

### Effect on tumor suppressor pathways

Tumor suppressor genes protect normal cells by preventing the oncogenic transformation into cancer cells [113]. In general, tumor suppressors such as p53, PTEN, Rb proteins help prevent the damage to DNA caused by the high intensity radiation, toxic chemicals such as dyes and infections especially by viruses [100]. In addition, tumor suppressor proteins also help in the scavenging of damaged cells through apoptosis [113]. Proof-of-principle studies expressing the tumor suppressors, in the cells where the expression of these genes is lost, due to the chromosomal deletions or mutational inactivation; have confirmed the anti-oncogenic role of these proteins [76]. Therefore, compounds that can trigger the expression of tumor suppressor proteins are likely to inhibit the development and transformation of cells into cancerous ones [114]**.** In a recent study, caffeic acid phenyl esters has been shown to arrest castration resistant prostate cancer (CRPC) cells in the G0/G1 phase of the cell cycle by upregulating p53, p21 and p27 proteins [115]. Confirming this data, knocking down these tumor suppressors using siRNA attenuated the efficacy of caffeic acid phenyl esters [115]. Similarly, an increase in the expression of p53 was observed when cervical cancer cells were exposed to caffeic acid [116]. Likewise, caffeic acid phenyl ester and caffeic acid octyl ester could also inhibit the growth of ME180 cervical cancer cells [117]. Another phenolic compound that has been demonstrated to upregulate tumor suppressors is ferulic acid. Ferulic acid enhanced the levels of p53 and p21 mRNA when transformed keratinocyte cell line HaCaT is exposed to UVB radiation [118]. Even the benzoic acid derivatives such as protocatechuic acid also induced the levels of p21 and p27 in leiomyoma cells. However, no major changes were reported, by this study, in the expression of p53 [119]. In summary, both cinnamic acid and benzoic acid derivatives can induce cell cycle arrest by inducing the expressions of the tumor suppressor genes and thereby inhibit the tumor cell proliferation.

### Effect on cytokine and cell differentiation pathways

Cytokines such as interferons, interleukins, tumor necrosis factor, lymphokines are low molecular weight proteins involved in cell signaling, development and immune responses [120]. Uncontrolled release of cytokines during oxidative stress or chronic inflammation induces malignancy in normal cells [121]. Cancer cells responds to the cytokines released by the host cells to induce cell growth, inhibit apoptosis and induce metastasis [122]. For example, cytokine IL-21 activates the STAT3 pathways by upregulating the Myc-c expression [123]. Myc-c is a key regulatory gene and is mutated in 70% cancers [114]. Mutated Myc-c fails to control the differentiation of cells [124]. Moreover, Myc-c gene is involved in cell cycle regulation, differentiation, metabolism and cell growth. Hence, molecules that promote anti-proliferative cytokines expression are important anticancer agents. Protocatechuic acid is one such molecule known to lower the levels of oncogenic IL-6 and IL-8 in a dose dependent fashion in cancer cell lines representing the breast, prostate, cervix, lung and liver [47]. Similar way, ferulic acid blocks the secretion of IL-6 and TNF-α in HaCaT cells [118]. Other phenolic compounds such as p-coumaric acid and caffeic acid have downregulated the expression of Myc-c, CCNB1, CCNA2 in CaCo2, HCT-116/SW480 cells leading to inhibition of cellular differentiation and proliferation [125].

### Effect on matrix metalloproteinases

Matrix metalloproteinases are endopeptidases capable of degrading the extracellular matrix [126]. Tumor cells express high levels of MMP, which degrade the extracellular matrix to promote tumor invasion and metastasis [127]. Thus molecules that inhibit MMPs can successfully inhibit the tumor growth and proliferation. A recent study demonstrated that protocatechuic acid suppressed the matrix metalloproteinase (MMP-2 and MMP-9) [109]. Similarly, ferulic acid inhibited the MMP2 and MMP9 in HUVEC and melanoma cells thereby inhibited the metastasis and angiogenesis of these cells [88]. CAA and CAPE, two other cinnamic acid derivatives, could selectively suppress the MMP-9 and MMP-2 activities to prevent hepatoma cells’ growth and metastasis [86]. Hence, phenolic compounds with MMP inhibitory properties could be considered for inhibiting the metastatic spread of tumor cells. However, further studies are warranted to conclusively and experimentally prove this data in preclinical animal models and clinical testing in humans.

## Adverse effects of phenolic compounds

Accumulating evidences from the literature suggests that the phenolic compounds despite their enormous health beneficial effects also possess adverse effects, which are primarily due to: (a) poor permeability if they exist as free acids; (b) the ability to transform normal cells in to cancer cells thereby induce tumors in certain cases; (c) induce systemic toxicity when administered in excess. Moreover, since phenolic compounds are known to scavenge reactive oxygen species, which are required for some of the key biological processes such as prevention of infections and inflammatory reactions, administering phenolic compounds to treat cancers might affect these processes and leads to infections and unusual inflammatory reactions [128].

### Toxicity

Although phenolic acids are well known for their antioxidant activity, they also act as pro-oxidants in presence of redox active metals. This pro-oxidant effect leads to deleterious effects on DNA, proteins and lipids [128]. Further, at higher concentrations phenolic acids can induce blisters and harm healthy cells. Thus the dose and exposure time are critical while administering phenolic acids [129]. Development of nanoformulations is likely to help reduce these off target effects caused by the phenolic acids.

### Tumorogenicity

Despite the antitumor effects of phenolic acids, few reports have shown tumor inducing properties [130]. For instance, a study conducted by Miller et al in 2001 showed estrogenic activity of phenolic acids, which lead to the development of breast cancer [130]. In addition a study in animal models has shown that 2% Caffeic acid administered through diet could induce 57% forestomach squamous cell carcinomas in female F344 rats and B6C3F1 mice [74]. Further, since some phenolic acids at lower doses are known to activate oncogenes, their administration is likely to induce tumors growth [131]. For example, Nrf-2, a key redox regulator, is activated by gallic acid and causes the induction of chemoresistance [131]. Similarly, a study conducted by Ashok et al 2015, also showed the ability of gallic acid to upregulate Nrf-2 activity in mice models [132]. Few recent studies have also demonstrated the ability of cinnamic acids to upregulate Nrf2. For instance, a study has shown that caffeic acid phenyl ester and ferulic acid activated the Nrf-2 expression in cancer cell lines [133, 134]. Despite these adverse effects, the research and use of phenolic compounds for treating cancers continues due to the predominance of health beneficial effects compared to adverse events. In addition, due to recent advancements in drug delivery and treatment options, even the adverse effects of phenolic compounds could also be easily managed, making the phenolic compounds as the choice of treatment for cancers.

## Conclusions

Even though plant derived phenolic compounds have been studied extensively for inhibiting tumor cell growth in vitro and in vivo, many gaps still persist that require additional studies. For example, cinnamic acid derivatives, caffeic acid and ferulic acid are known to down modulate survival and proliferation signaling cascades such as PI3K-Akt and MAPK pathways, respectively. However, it is not fully known how these cinnamic acid derivatives are retarding these signaling cascades. Are they binding to Akt or Raf proteins? Or are they inhibiting the upstream kinases/binding proteins? Therefore, additional studies are required to address these concerns. Furthermore, experimental data is required to address variations in treatment response among individuals who are consuming phenolic acid rich diet. Additional studies are also required to improve the therapeutic efficacy and tumor cell selectivity of phenolic compounds. Since benzoic and cinnamic acid derivatives are effective only at higher doses (about 10–20 mg/kg body weight) strategies to reduce the dose and toxicity are urgently required. Hence, future studies should focus on developing targeted nanoformulations loaded with anti-tumor phenolic compounds. In conclusion, the review summarized: (a) key structural features of phenolic acids required for exhibiting anti-cancer activity; (b) the recently published data highlighting the beneficial effects of biotransformation of phenolic acids by gut microbiota; (c) the mechanisms of action(s) of phenolic acids leading to the inhibition of cancer cell proliferation and migration; and (d) the preclinical data assessing the pharmacological behavior including the safety and anti-cancer activity of phenolic acids.

## Abbreviations

AKT: Protein kinase B
APC: Adenomatous polyposis coli
BA: Benzoic acid
Bax: BCl_2_ Associated X protein
BCl_2_: B cell lymphoma 2
CA: Cinnamic acid
CAA: Caffeic acid
CAOE: Caffeic acid octyl ester
CAPE: Caffeic acid phenyl ester
CAPP: Caffeic acid phenyl propyl ester
EGF: Epidermal Growth Factor
EMT: Epithelial-Mesenchymal Transition
ERK: Extracellular signal regulated kinase
FA: Ferulic acid
IC_50_: Inhibitory concentration
IL-21, IL-6, IL-8: Interleukins
LD_50_: Lethal dose
LPO: Lactoperoxidase
MAPK: Mitogen activated protein kinase
MCT: Monocarboxylic acid transporters
MMP-2 and MMP-9: Matrix metalloproteinase
MPO: Myeloperoxidase
m-TOR: Mechanistic target of rapamycin
MTT: 3-(4, 5-Dimethylthiazol-2-yl)-2, 5-Diphenyltetrazolium Bromide
NF-kβ: Nuclear factor kappa-light-chain-enhancer of activated B cells
PCA: Protocatechuic acid
p-CoA: para- Coumaric acid
PI3K: Phosphatidylinositol 3-kinase
PTEN: Phosphatase and tensin homolog deleted on chromosome-10
ROS: Reactive oxygen species
SCID: Severe combined immunodeficiency
siRNA: Small interfering RNA
SRB: Sulphorhodamine B
STAT: Signal transducer and activator of transcription
VEGF: Vascular Endothelial Growth Factor

## References

[1] Russell W, Duthie G (2011). Plant secondary metabolites and gut health: the case for phenolic acids. Proc Nutr Soc.

[2] Tsao R (2010). Chemistry and biochemistry of dietary polyphenols. Nutrients.

[3] Huang WY, Cai YZ, Zhang Y (2010). Natural phenolic compounds from medicinal herbs and dietary plants: potential use for cancer prevention. Nutr Cancer.

[4] Arts IC, Hollman PC (2005). Polyphenols and disease risk in epidemiologic studies. Am J Clin Nutr.

[5] Lambert JD, Hong J, Yang GY, Liao J, Yang CS (2005). Inhibition of carcinogenesis by polyphenols: evidence from laboratory investigations. Am J Clin Nutr.

[6] Tzin V, Galili G (2010). The biosynthetic pathways for shikimate and aromatic amino acids in Arabidopsis thaliana. Arabidopsis Book.

[7] de Lourdes Reis Giada M. Food Phenolic Compounds: Main Classes, Sources and Their Antioxidant Power. In: Jose Antonio Morales-Gonzalez, Editors. Oxidative Stress and Chronic Degenerative Diseases - A Role for Antioxidants. InTech; 2013. p. 87–112.

[8] Yang CS, Landau JM, Huang MT, Newmark HL (2001). Inhibition of carcinogenesis by dietary polyphenolic compounds. Annu Rev Nutr.

[9] Khan NS, Hadi SM (1998). Structural features of tannic acid important for DNA degradation in the presence of Cu(II). Mutagenesis.

[10] De P, Baltas M, Bedos-Belval F (2011). Cinnamic acid derivatives as anticancer agents-a review. Curr Med Chem.

[11] Cooper GM (2000). The cell: a molecular approach.

[12] Pandey KB, Rizvi SI (2009). Plant polyphenols as dietary antioxidants in human health and disease. Oxid Med Cell Longev.

[13] Kumar S, Pandey AK (2013). Chemistry and biological activities of flavonoids: an overview. ScientificWorldJournal.

[14] Cheynier V (2005). Polyphenols in foods are more complex than often thought. Am J Clin Nutr.

[15] Heim KE, Tagliaferro AR, Bobilya DJ (2002). Flavonoid antioxidants: chemistry, metabolism and structure-activity relationships. J Nutr Biochem.

[16] Akk A (2009). Response of plant parts and age on distribution of secondary metabolities on plants in Quetta. Pak J Bot.

[17] Dai J, Mumper RJ (2010). Plant phenolics: extraction, analysis and their antioxidant and anticancer properties. Molecules.

[18] Ververidis F, Trantas E, Douglas C, Vollmer G, Kretzschmar G, Panopoulos N (2007). Biotechnology of flavonoids and other phenylpropanoid-derived natural products. Part II: Reconstruction of multienzyme pathways in plants and microbes. Biotechnol J.

[19] Spencer JP, Abd El Mohsen MM, Minihane AM, Mathers JC (2008). Biomarkers of the intake of dietary polyphenols: strengths, limitations and application in nutrition research. Br J Nutr.

[20] Rossetto M, Lante A, Vanzani P, Spettoli P, Scarpa M, Rigo A (2005). Red chicories as potent scavengers of highly reactive radicals: a study on their phenolic composition and peroxyl radical trapping capacity and efficiency. J Agric Food Chem.

[21] Kivilompolo M, Oburka V, Hyotylainen T (2007). Comparison of GC-MS and LC-MS methods for the analysis of antioxidant phenolic acids in herbs. Anal Bioanal Chem.

[22] Perez-Jimenez J, Neveu V, Vos F, Scalbert A (2010). Identification of the 100 richest dietary sources of polyphenols: an application of the Phenol-Explorer database. Eur J Clin Nutr.

[23] Valdez-Morales M, Espinosa-Alonso LG, Espinoza-Torres LC, Delgado-Vargas F, Medina-Godoy S (2014). Phenolic content and antioxidant and antimutagenic activities in tomato peel, seeds, and byproducts. J Agric Food Chem.

[24] Akyol H, Riciputi Y, Capanoglu E, Caboni MF, Verardo V. Phenolic compounds in the potato and its byproducts: An overview. Int J Mol Sci. 2016;17:835. doi:10.3390/ijms17060835. 10.3390/ijms17060835 PMC4926369 27240356

[25] Liu RJ, Zhu H, Ding L, Shakya S, Yang ZL, Cheng L (2013). [Study on pharmacokinetics of asperosaponin VI and its active metabolite in rats]. Zhongguo Zhong Yao Za Zhi.

[26] Martinez-Valverde I, Periago MJ, Ros G (2000). [Nutritional importance of phenolic compounds in the diet]. Arch Latinoam Nutr.

[27] Crozier A, Jaganath IB, Clifford MN (2009). Dietary phenolics: chemistry, bioavailability and effects on health. Nat Prod Rep.

[28] Davalli P, Rizzi F, Caporali A, Pellacani D, Davoli S, Bettuzzi S, Brausi M, D’Arca D (2012). Anticancer activity of green tea polyphenols in prostate gland. Oxid Med Cell Longev.

[29] Kandaswami C, Lee LT, Lee PP, Hwang JJ, Ke FC, Huang YT, Lee MT (2005). The antitumor activities of flavonoids. In Vivo.

[30] Mursu J, Nurmi T, Tuomainen TP, Ruusunen A, Salonen JT, Voutilainen S (2007). The intake of flavonoids and carotid atherosclerosis: the Kuopio Ischaemic Heart Disease Risk Factor Study. Br J Nutr.

[31] Letenneur L, Proust-Lima C, Le Gouge A, Dartigues JF, Barberger-Gateau P (2007). Flavonoid intake and cognitive decline over a 10-year period. Am J Epidemiol.

[32] Marin L, Miguelez EM, Villar CJ, Lombo F (2015). Bioavailability of dietary polyphenols and gut microbiota metabolism: antimicrobial properties. Biomed Res Int.

[33] Saha P, Yeoh BS, Singh R, Chandrasekar B, Vemula PK, Haribabu B, Vijay-Kumar M, Jala VR (2016). Gut microbiota conversion of dietary ellagic acid into bioactive phytoceutical urolithin a inhibits heme peroxidases. PLoS One.

[34] Vyas U, Ranganathan N (2012). Probiotics, prebiotics, and synbiotics: gut and beyond. Gastroenterol Res Pract.

[35] Ghasemzadeh A, Jaafar HZ (2013). Profiling of phenolic compounds and their antioxidant and anticancer activities in pandan (Pandanus amaryllifolius Roxb.) extracts from different locations of Malaysia. BMC Complement Altern Med.

[36] de Oliveira CB, Comunello LN, Maciel ES, Giubel SR, Bruno AN, Chiela EC, Lenz G, Gnoatto SC, Buffon A, Gosmann G (2013). The inhibitory effects of phenolic and terpenoid compounds from Baccharis trimera in Siha cells: differences in their activity and mechanism of action. Molecules.

[37] Senawong T, Khaopha S, Misunaa S, Komaikula J, Senawonga G, Wongphakhama P, Yunchalard S (2014). Phenolic acid composition and anticancer activity against human cancer cell lines of the commercially available fermentation products of Houttuynia cordata. Sci Asia.

[38] Chen HM, Wu YC, Chia YC, Chang FR, Hsu HK, Hsieh YC, Chen CC, Yuan SS (2009). Gallic acid, a major component of Toona sinensis leaf extracts, contains a ROS-mediated anti-cancer activity in human prostate cancer cells. Cancer Lett.

[39] Kurata R, Adachi M, Yamakawa O, Yoshimoto M (2007). Growth suppression of human cancer cells by polyphenolics from sweetpotato (Ipomoea batatas L.) leaves. J Agric Food Chem.

[40] Lee YJ, Liao PH, Chen WK, Yang CY (2000). Preferential cytotoxicity of caffeic acid phenethyl ester analogues on oral cancer cells. Cancer Lett.

[41] Chen M, Meng H, Zhao Y, Chen F, Yu S (2015). Antioxidant and in vitro anticancer activities of phenolics isolated from sugar beet molasses. BMC Complement Altern Med.

[42] Shahrzad S, Aoyagi K, Winter A, Koyama A, Bitsch I (2001). Pharmacokinetics of gallic acid and its relative bioavailability from tea in healthy humans. J Nutr.

[43] Ho HH, Chang CS, Ho WC, Liao SY, Lin WL, Wang CJ (2013). Gallic acid inhibits gastric cancer cells metastasis and invasive growth via increased expression of RhoB, downregulation of AKT/small GTPase signals and inhibition of NF-kappaB activity. Toxicol Appl Pharmacol.

[44] Lin HH, Chen JH, Chou FP, Wang CJ (2011). Protocatechuic acid inhibits cancer cell metastasis involving the down-regulation of Ras/Akt/NF-kappaB pathway and MMP-2 production by targeting RhoB activation. Br J Pharmacol.

[45] Kassi E, Chinou I, Spilioti E, Tsiapara A, Graikou K, Karabournioti S, Manoussakis M, Moutsatsou P (2014). A monoterpene, unique component of thyme honeys, induces apoptosis in prostate cancer cells via inhibition of NF-kappaB activity and IL-6 secretion. Phytomedicine.

[46] Seidel C, Schnekenburger M, Dicato M, Diederich M (2014). Antiproliferative and proapoptotic activities of 4-hydroxybenzoic acid-based inhibitors of histone deacetylases. Cancer Lett.

[47] Yin MC, Lin CC, Wu HC, Tsao SM, Hsu CK (2009). Apoptotic effects of protocatechuic acid in human breast, lung, liver, cervix, and prostate cancer cells: potential mechanisms of action. J Agric Food Chem.

[48] Wang LC, Chu KH, Liang YC, Lin YL, Chiang BL (2010). Caffeic acid phenethyl ester inhibits nuclear factor-kappaB and protein kinase B signalling pathways and induces caspase-3 expression in primary human CD4+ T cells. Clin Exp Immunol.

[49] Fukuda M, Kobayashi K, Hirono Y, Miyagawa M, Ishida T, Ejiogu EC, Sawai M, Pinkerton KE, Takeuchi M (2011). Jungle honey enhances immune function and antitumor activity. Evid Based Complement Alternat Med.

[50] Samarghandian S, Afshari JT, Davoodi S (2011). Honey induces apoptosis in renal cell carcinoma. Pharmacogn Mag.

[51] Lee YJ, Kuo HC, Chu CY, Wang CJ, Lin WC, Tseng TH (2003). Involvement of tumor suppressor protein p53 and p38 MAPK in caffeic acid phenethyl ester-induced apoptosis of C6 glioma cells. Biochem Pharmacol.

[52] Jaganathan SK, Supriyanto E, Mandal M (2013). Events associated with apoptotic effect of p-Coumaric acid in HCT-15 colon cancer cells. World J Gastroenterol.

[53] Nagaoka T, Banskota AH, Tezuka Y, Saiki I, Kadota S (2002). Selective antiproliferative activity of caffeic acid phenethyl ester analogues on highly liver-metastatic murine colon 26-L5 carcinoma cell line. Bioorg Med Chem.

[54] Hanahan D, Weinberg RA (2011). Hallmarks of cancer: the next generation. Cell.

[55] Shapiro GI, Harper JW (1999). Anticancer drug targets: cell cycle and checkpoint control. J Clin Invest.

[56] Niero EL, Machado-Santelli GM (2013). Cinnamic acid induces apoptotic cell death and cytoskeleton disruption in human melanoma cells. J Exp Clin Cancer Res.

[57] Guo H, Li M, Chen P, Blake DJ, Kong X, Hao X, Niu R, Zhang N (2011). 4-Methyl-3-nitro-benzoic acid, a migration inhibitor, prevents breast cancer metastasis in SCID mice. Cancer Lett.

[58] Murad LD, Soares Nda C, Brand C, Monteiro MC, Teodoro AJ (2015). Effects of caffeic and 5-caffeoylquinic acids on cell viability and cellular uptake in human colon adenocarcinoma cells. Nutr Cancer.

[59] Rajendra Prasad N, Karthikeyan A, Karthikeyan S, Reddy BV (2011). Inhibitory effect of caffeic acid on cancer cell proliferation by oxidative mechanism in human HT-1080 fibrosarcoma cell line. Mol Cell Biochem.

[60] Fahrioglu U, Dodurga Y, Elmas L, Secme M (2016). Ferulic acid decreases cell viability and colony formation while inhibiting migration of MIA PaCa-2 human pancreatic cancer cells in vitro. Gene.

[61] Zhao B, Hu M (2013). Gallic acid reduces cell viability, proliferation, invasion and angiogenesis in human cervical cancer cells. Oncol Lett.

[62] Sourani Z (2015). The effect of gallic acid on Jurkat cell line. J HerbMed Pharmacol.

[63] Pontiki E, Hadjipavlou-Litina D, Litinas K, Geromichalos G (2014). Novel cinnamic acid derivatives as antioxidant and anticancer agents: design, synthesis and modeling studies. Molecules.

[64] Elmore S (2007). Apoptosis: a review of programmed cell death. Toxicol Pathol.

[65] Pirzad G, Jafari M, Tavana S, Sadrayee H, Ghavami S, Shajiei A, Ghanei M (2010). The role of Fas-FasL signaling pathway in induction of apoptosis in patients with sulfur mustard-induced chronic bronchiolitis. J Toxicol.

[66] Rieger AM, Nelson KL, Konowalchuk JD, Barreda DR. Modified Annexin V/Propidium Iodide Apoptosis Assay For Accurate Assessment of Cell Death. Journal of Visualized Experiments: JoVE. 2011;50;2597. doi:10.3791/2597. 10.3791/2597 PMC3169266 21540825

[67] Annamalai P, Thayman M, Rajan S, Raman LS, Ramasubbu S, Perumal P (2015). Ethyl acetate extract from marine sponge Hyattella cribriformis exhibit potent anticancer activity by promoting tubulin polymerization as evidenced mitotic arrest and induction of apoptosis. Pharmacogn Mag.

[68] Clark AG, Vignjevic DM (2015). Modes of cancer cell invasion and the role of the microenvironment. Curr Opin Cell Biol.

[69] Zhang X, Lin D, Jiang R, Li H, Wan J, Li H (2016). Ferulic acid exerts antitumor activity and inhibits metastasis in breast cancer cells by regulating epithelial to mesenchymal transition. Oncol Rep.

[70] Fraser SP, Hemsley F, Djamgoz MB (2016). Caffeic acid phenethyl ester: Inhibition of metastatic cell behaviours via voltage-gated sodium channel in human breast cancer in vitro. Int J Biochem Cell Biol.

[71] Andrikopoulos P, Fraser SP, Patterson L, Ahmad Z, Burcu H, Ottaviani D, Diss JK, Box C, Eccles SA, Djamgoz MB (2011). Angiogenic functions of voltage-gated Na + Channels in human endothelial cells: modulation of vascular endothelial growth factor (VEGF) signaling. J Biol Chem.

[72] Peng CY, Yang HW, Chu YH, Chang YC, Hsieh MJ, Chou MY, Yeh KT, Lin YM, Yang SF, Lin CW (2012). Caffeic Acid phenethyl ester inhibits oral cancer cell metastasis by regulating matrix metalloproteinase-2 and the mitogen-activated protein kinase pathway. Evid Based Complement Alternat Med.

[73] Habauzit V, Morand C (2012). Evidence for a protective effect of polyphenols-containing foods on cardiovascular health: an update for clinicians. Ther Adv Chronic Dis.

[74] Hirose M, Fukushima S, Shirai T, Hasegawa R, Kato T, Tanaka H, Asakawa E, Ito N (1990). Stomach carcinogenicity of caffeic acid, sesamol and catechol in rats and mice. Jpn J Cancer Res.

[75] Kakkar S, Bais S (2014). A review on protocatechuic Acid and its pharmacological potential. ISRN Pharmacol.

[76] Niho N, Shibutani M, Tamura T, Toyoda K, Uneyama C, Takahashi N, Hirose M (2001). Subchronic toxicity study of gallic acid by oral administration in F344 rats. Food Chem Toxicol.

[77] Rajalakshmi K, Devaraj H, Niranjali Devaraj S (2001). Assessment of the no-observed-adverse-effect level (NOAEL) of gallic acid in mice. Food Chem Toxicol.

[78] Pei K, Ou J, Huang J, Ou S (2016). p-Coumaric acid and its conjugates: dietary sources, pharmacokinetic properties and biological activities. J Sci Food Agric.

[79] Turner PV, Brabb T, Pekow C, Vasbinder MA (2011). Administration of substances to laboratory animals: routes of administration and factors to consider. J Am Assoc Lab Anim Sci.

[80] Konishi Y, Hitomi Y, Yoshioka E (2004). Intestinal absorption of p-coumaric and gallic acids in rats after oral administration. J Agric Food Chem.

[81] Kern SM, Bennett RN, Mellon FA, Kroon PA, Garcia-Conesa MT (2003). Absorption of hydroxycinnamates in humans after high-bran cereal consumption. J Agric Food Chem.

[82] Konishi Y, Kobayashi S, Shimizu M (2003). Transepithelial transport of p-coumaric acid and gallic acid in Caco-2 cell monolayers. Biosci Biotechnol Biochem.

[83] Adam A, Crespy V, Levrat-Verny MA, Leenhardt F, Leuillet M, Demigne C, Remesy C (2002). The bioavailability of ferulic acid is governed primarily by the food matrix rather than its metabolism in intestine and liver in rats. J Nutr.

[84] D’Archivio M, Filesi C, Di Benedetto R, Gargiulo R, Giovannini C, Masella R (2007). Polyphenols, dietary sources and bioavailability. Ann Ist Super Sanita.

[85] Chiang EP, Tsai SY, Kuo YH, Pai MH, Chiu HL, Rodriguez RL, Tang FY (2014). Caffeic acid derivatives inhibit the growth of colon cancer: involvement of the PI3-K/Akt and AMPK signaling pathways. PLoS One.

[86] Chung TW, Moon SK, Chang YC, Ko JH, Lee YC, Cho G, Kim SH, Kim JG, Kim CH (2004). Novel and therapeutic effect of caffeic acid and caffeic acid phenyl ester on hepatocarcinoma cells: complete regression of hepatoma growth and metastasis by dual mechanism. FASEB J.

[87] Onori P, DeMorrow S, Gaudio E, Franchitto A, Mancinelli R, Venter J, Kopriva S, Ueno Y, Alvaro D, Savage J (2009). Caffeic acid phenethyl ester decreases cholangiocarcinoma growth by inhibition of NF-kappaB and induction of apoptosis. Int J Cancer.

[88] Yang GW, Jiang JS, Lu WQ (2015). Ferulic acid exerts anti-angiogenic and anti-tumor activity by targeting fibroblast growth factor receptor 1-mediated angiogenesis. Int J Mol Sci.

[89] Liang CZ, Zhang X, Li H, Tao YQ, Tao LJ, Yang ZR, Zhou XP, Shi ZL, Tao HM (2012). Gallic acid induces the apoptosis of human osteosarcoma cells in vitro and in vivo via the regulation of mitogen-activated protein kinase pathways. Cancer Biother Radiopharm.

[90] Raina K, Rajamanickam S, Deep G, Singh M, Agarwal R, Agarwal C (2008). Chemopreventive effects of oral gallic acid feeding on tumor growth and progression in TRAMP mice. Mol Cancer Ther.

[91] Zhu B, Shang B, Li Y, Zhen Y (2016). Inhibition of histone deacetylases by trans-cinnamic acid and its antitumor effect against colon cancer xenografts in athymic mice. Mol Med Rep.

[92] Murphy MP (2009). How mitochondria produce reactive oxygen species. Biochem J.

[93] Ray PD, Huang BW, Tsuji Y (2012). Reactive oxygen species (ROS) homeostasis and redox regulation in cellular signaling. Cell Signal.

[94] Rahman K (2007). Studies on free radicals, antioxidants, and co-factors. Clin Interv Aging.

[95] Gill SS, Tuteja N (2010). Reactive oxygen species and antioxidant machinery in abiotic stress tolerance in crop plants. Plant Physiol Biochem.

[96] Nakabeppu Y, Tsuchimoto D, Ichinoe A, Ohno M, Ide Y, Hirano S, Yoshimura D, Tominaga Y, Furuichi M, Sakumi K (2004). Biological significance of the defense mechanisms against oxidative damage in nucleic acids caused by reactive oxygen species: from mitochondria to nuclei. Ann N Y Acad Sci.

[97] Benedetti S, Nuvoli B, Catalani S, Galati R (2015). Reactive oxygen species a double-edged sword for mesothelioma. Oncotarget.

[98] Nikitaki Z, Hellweg CE, Georgakilas AG, Ravanat JL (2015). Stress-induced DNA damage biomarkers: applications and limitations. Front Chem.

[99] Jaganathan SK (2012). Growth inhibition by caffeic acid, one of the phenolic constituents of honey, in HCT 15 colon cancer cells. ScientificWorldJournal.

[100] Lodish HBA, Zipursky SL (2000). Molecular cell biology.

[101] Davies H, Bignell GR, Cox C, Stephens P, Edkins S, Clegg S, Teague J, Woffendin H, Garnett MJ, Bottomley W (2002). Mutations of the BRAF gene in human cancer. Nature.

[102] Leicht DT, Balan V, Kaplun A, Singh-Gupta V, Kaplun L, Dobson M, Tzivion G (1773). Raf kinases: function, regulation and role in human cancer. Biochim Biophys Acta.

[103] Wajapeyee N, Serra RW, Zhu X, Mahalingam M, Green MR (2008). Oncogenic BRAF induces senescence and apoptosis through pathways mediated by the secreted protein IGFBP7. Cell.

[104] Cheung M, Sharma A, Madhunapantula SV, Robertson GP (2008). Akt3 and mutant V600E B-Raf cooperate to promote early melanoma development. Cancer Res.

[105] Aquilato A, Lopez V, Doonan B, Hsieh T-C, Pinto JT, Wu E, Wu JM (2014). RAF mutation in melanoma and DietaryPolyphenols as adjunctive treatment strategy. Polyphenols Hum Health Dis.

[106] Lamoral-Theys D, Pottier L, Dufrasne F, Neve J, Dubois J, Kornienko A, Kiss R, Ingrassia L (2010). Natural polyphenols that display anticancer properties through inhibition of kinase activity. Curr Med Chem.

[107] Kang NJ, Lee KW, Kim BH, Bode AM, Lee HJ, Heo YS, Boardman L, Limburg P, Lee HJ, Dong Z (2011). Coffee phenolic phytochemicals suppress colon cancer metastasis by targeting MEK and TOPK. Carcinogenesis.

[108] Kim SR, Jung YR, Kim DH, An HJ, Kim MK, Kim ND, Chung HY (2014). Caffeic acid regulates LPS-induced NF-kappaB activation through NIK/IKK and c-Src/ERK signaling pathways in endothelial cells. Arch Pharm Res.

[109] Tsao SM, Hsia TC, Yin MC (2014). Protocatechuic acid inhibits lung cancer cells by modulating FAK, MAPK, and NF-kappaB pathways. Nutr Cancer.

[110] Pramanik KC, Kudugunti SK, Fofaria NM, Moridani MY, Srivastava SK (2013). Caffeic acid phenethyl ester suppresses melanoma tumor growth by inhibiting PI3K/AKT/XIAP pathway. Carcinogenesis.

[111] Lin HP, Jiang SS, Chuu CP (2012). Caffeic acid phenethyl ester causes p21 induction, Akt signaling reduction, and growth inhibition in PC-3 human prostate cancer cells. PLoS One.

[112] Tsai CM, Yen GC, Sun FM, Yang SF, Weng CJ (2013). Assessment of the anti-invasion potential and mechanism of select cinnamic acid derivatives on human lung adenocarcinoma cells. Mol Pharm.

[113] Guo XE, Ngo B, Modrek AS, Lee WH (2014). Targeting tumor suppressor networks for cancer therapeutics. Curr Drug Targets.

[114] van Heemst D, Mooijaart SP, Beekman M, Schreuder J, de Craen AJ, Brandt BW, Slagboom PE, Westendorp RG, Long Life study g (2005). Variation in the human TP53 gene affects old age survival and cancer mortality. Exp Gerontol.

[115] Lin HP, Lin CY, Huo C, Hsiao PH, Su LC, Jiang SS, Chan TM, Chang CH, Chen LT, Kung HJ (2015). Caffeic acid phenethyl ester induced cell cycle arrest and growth inhibition in androgen-independent prostate cancer cells via regulation of Skp2, p53, p21Cip1 and p27Kip1. Oncotarget.

[116] Chang WC, Hsieh CH, Hsiao MW, Lin WC, Hung YC, Ye JC (2010). Caffeic acid induces apoptosis in human cervical cancer cells through the mitochondrial pathway. Taiwan J Obstet Gynecol.

[117] Hung MW, Shiao MS, Tsai LC, Chang GG, Chang TC (2003). Apoptotic effect of caffeic acid phenethyl ester and its ester and amide analogues in human cervical cancer ME180 cells. Anticancer Res.

[118] Lin X-F (2010). Anticarcinogenic effect of ferulic acid on ultraviolet-B irradiated human keratinocyte HaCaT cells. J Med Plants Res.

[119] Shon W-K (2008). Induction of apoptosis by Hibiscus protocatechuic acid in human uterine leiomyoma cells. Korean J Gynecol Oncol.

[120] Dinarello CA (2000). Proinflammatory cytokines. Chest.

[121] Zamarron BF, Chen W (2011). Dual roles of immune cells and their factors in cancer development and progression. Int J Biol Sci.

[122] Dranoff G (2004). Cytokines in cancer pathogenesis and cancer therapy. Nat Rev Cancer.

[123] Sarosiek KA, Malumbres R, Nechushtan H, Gentles AJ, Avisar E, Lossos IS (2010). Novel IL-21 signaling pathway up-regulates c-Myc and induces apoptosis of diffuse large B-cell lymphomas. Blood.

[124] Nilsson JA, Cleveland JL (2003). Myc pathways provoking cell suicide and cancer. Oncogene.

[125] Xiang D, Wang D, He Y, Xie J, Zhong Z, Li Z, Xie J (2006). Caffeic acid phenethyl ester induces growth arrest and apoptosis of colon cancer cells via the beta-catenin/T-cell factor signaling. Anticancer Drugs.

[126] Zhu JY, Pang ZJ, Yu YH (2012). Regulation of trophoblast invasion: the role of matrix metalloproteinases. Rev Obstet Gynecol.

[127] Kessenbrock K, Plaks V, Werb Z (2010). Matrix metalloproteinases: regulators of the tumor microenvironment. Cell.

[128] Kyselova Z (2011). Toxicological aspects of the use of phenolic compounds in disease prevention. Interdiscip Toxicol.

[129] Vermerris W, Nicholson R. Phenolic compounds and their effects on human health. In: Vermerris W, Nicholson R, editors. Phenolic compound biochemistry. Springer Science; 2006. p. 235–255.

[130] Miller D, Wheals BB, Beresford N, Sumpter JP (2001). Estrogenic activity of phenolic additives determined by an in vitro yeast bioassay. Environ Health Perspect.

[131] Sporn MB, Liby KT (2012). NRF2 and cancer: the good, the bad and the importance of context. Nat Rev Cancer.

[132] Pandurangan AK, Mohebali N, Norhaizan ME, Looi CY (2015). Gallic acid attenuates dextran sulfate sodium-induced experimental colitis in BALB/c mice. Drug Des Devel Ther.

[133] Kim H, Kim W, Yum S, Hong S, Oh JE, Lee JW, Kwak MK, Park EJ, Na DH, Jung Y (2013). Caffeic acid phenethyl ester activation of Nrf2 pathway is enhanced under oxidative state: structural analysis and potential as a pathologically targeted therapeutic agent in treatment of colonic inflammation. Free Radic Biol Med.

[134] Ma ZC, Hong Q, Wang YG, Liang QD, Tan HL, Xiao CR, Tang XL, Shao S, Zhou SS, Gao Y (2011). Ferulic acid induces heme oxygenase-1 via activation of ERK and Nrf2. Drug Discov Ther.

[135] Pacheco-Palencia LA, Mertens-Talcott S, Talcott ST (2008). Chemical composition, antioxidant properties, and thermal stability of a phytochemical enriched oil from Acai (Euterpe oleracea Mart.). J Agric Food Chem.

[136] Vogt T (2010). Phenylpropanoid biosynthesis. Mol Plant.

[137] Budavari S (1996). The Merck index: an encyclopedia of chemicals, drugs, and biologicals.

[138] Hernanz D, Nunez V, Sancho AI, Faulds CB, Williamson G, Bartolome B (2001). Gomez-Cordoves C Hydroxycinnamic acids and ferulic acid dehydrodimers in barley and processed barley. J Agric Food Chem.

[139] Mao WSM, Berenbaum MR (2013). Honey constituents up-regulate detoxification and immunity genes in the western honey bee Apis mellifera. Proc Natl Acad Sci U S A.

[140] Li CY, Lee EJ, Wu TS (2004). Antityrosinase principles and constituents of the petals of Crocus sativus. J Nat Prod.

[141] Khadem S, Marles RJ (2010). Monocyclic phenolic acids; hydroxy- and polyhydroxybenzoic acids: occurrence and recent bioactivity studies. Molecules.

[142] Quinde-Axtell Z, Baik BK (2006). Phenolic compounds of barley grain and their implication in food product discoloration. J Agric Food Chem.

[143] Onakpoya I, Terry R, Ernst E (2011). The use of green coffee extract as a weight loss supplement: a systematic review and meta-analysis of randomised clinical trials. Gastroenterol Res Pract.

[144] Panizzi LS, Luisa M (1954). Constitution of cynarine, the active principle of the artichoke. Nature.

[145] Sanderson JT, Clabault H, Patton C, Lassalle-Claux G, Jean-Francois J, Pare AF, Hebert MJ, Surette ME, Touaibia M (2013). Antiproliferative, antiandrogenic and cytotoxic effects of novel caffeic acid derivatives in LNCaP human androgen-dependent prostate cancer cells. Bioorg Med Chem.

[146] Rzepecka-Stojko A, Kabala-Dzik A, Mozdzierz A, Kubina R, Wojtyczka RD, Stojko R, Dziedzic A, Jastrzebska-Stojko Z, Jurzak M, Buszman E, Stojko J (2015). Caffeic Acid phenethyl ester and ethanol extract of propolis induce the complementary cytotoxic effect on triple-negative breast cancer cell lines. Molecules.

[147] Shailasree S, Venkataramana M, Niranjana SR, Prakash HS (2015). Cytotoxic effect of p-Coumaric acid on neuroblastoma, N2a cell via generation of reactive oxygen species leading to dysfunction of mitochondria inducing apoptosis and autophagy. Mol Neurobiol.

[148] Chang MY, Shen YL (2014). Linalool exhibits cytotoxic effects by activating antitumor immunity. Molecules.

[149] Eroglu C, Secme M, Bagci G, Dodurga Y (2015). Assessment of the anticancer mechanism of ferulic acid via cell cycle and apoptotic pathways in human prostate cancer cell lines. Tumour Biol.

[150] Devi YP, Uma A, Narasu ML, Kalyani C (2014). Anticancer activity of gallic acid on cancer cell lines, HCT-15 and MDA MB 231. Int J Res Appl Nat Soc Sci.

